# Transactions of Illinois State Dental Society

**Published:** 1882-02

**Authors:** 


*Transactions of Illinois State Dental Society.*—17th Annual Session, held at Rock Island, May 10th, 1881. This report of Proceedings contains some very able and interesting articles from Drs. Dean, Harlan, Richards and others; and reflects credit upon the Publication Committee consisting of Drs. Noyes and Swain.

